# ASK1 in Cancer Cell Death: Insights from In Vitro and In Vivo Studies

**DOI:** 10.3390/cells15141282

**Published:** 2026-07-17

**Authors:** Eric J. O’Neill, Amanda Kornel, Emily C. Irwin, Evangelia Tsiani

**Affiliations:** Department of Health Sciences, Faculty of Applied Health Sciences, Brock University, St. Catharines, ON L2S 3A1, Canada

**Keywords:** cancer, ASK1, oxidative stress, apoptosis, NSCLC, colorectal cancer, breast cancer

## Abstract

**Highlights:**

**What are the main findings?**

- ASK1 activation is involved in anticancer effects of many natural and synthetic compounds across lung, gastrointestinal, and breast/gynecologic cancers, predominantly via ROS- or ER-stress-induced dissociation of inhibitory thioredoxin and downstream JNK/p38 signalling.
- In vivo xenograft studies confirm an association between ASK1 activation and decreased tumor burden.

**What are the implications of the main findings?**

- Compounds that disrupt Trx-ASK1 binding or elevate intracellular ROS may warrant prioritization in cancer drug development.

**Abstract:**

Apoptosis signal-regulating kinase 1 (ASK1) is a mitogen-activated protein kinase kinase kinase (MAP3K) involved in stress-induced apoptosis. Increasing evidence indicates that ASK1 activation contributes to the anticancer activity of numerous compounds, particularly those that induce oxidative or endoplasmic reticulum stress. This review summarizes studies demonstrating ASK1-dependent apoptosis in models of lung, breast and gynecologic, or gastrointestinal cancers following treatment with natural products, phytochemicals, and synthetic agents, focusing on mechanistic evidence linking ASK1 to downstream activation of the JNK and p38 MAPK pathways, mitochondrial dysfunction, and caspase-dependent cell death. Studies were selected based on direct experimental validation of ASK1 activation and involvement in the observed anticancer effects. Overall, this review supports ASK1 as a promising molecular target for the development of novel cancer treatment strategies.

## 1. Introduction

### 1.1. Cancer

Cancer is the second leading cause of premature death globally and the primary cause of premature death in both Canada and the USA [1]. In 2020, there were an estimated 19.3 million new cancer diagnoses and 10.0 million cancer deaths, globally [2]. While female breast cancer is the most diagnosed cancer worldwide followed by lung, colorectal, and prostate cancers, lung cancer accounts for the largest proportion of cancer deaths globally across sexes, followed by breast cancer in women and prostate cancer in men [2]. Interestingly, developed nations have a 2-fold to 3-fold greater incidence of cancer compared with developing nations, which may suggest lifestyle factors in developed countries contribute to cancer risk [2].

Hanahan and Weinberg [3] outline six core hallmarks which are generalizable to virtually all forms of cancer, although the specific cellular alterations underlying each hallmark vary between different cancers. These hallmarks include: sustained proliferative signalling, evasion of growth suppressors, activation of invasion and metastasis, enabling replicative immortality, inducing angiogenesis, and resisting apoptosis [3].

In normal cells, growth signals initiate the transition from a quiescent state to a proliferative one; however, cancer cells have acquired an ability to proliferate in the absence of typical growth signals [4]. Some cancer cells utilize autocrine proliferative signalling whereby neoplastic cells secrete growth factors that they respond to [5]. Alternatively, cancer cells may stimulate normal cells in the tumor stroma to secrete growth factors [6]. Deregulation of the growth factor receptors is another mechanism to achieve sustained proliferative signalling. Receptor overexpression can render cancer cells hyperresponsive to low levels of growth factor that would not normally trigger proliferation. Overexpression of epidermal growth factor receptor (EGFR) family proteins can be observed in many cancers, especially those of the breast, lungs, and ovaries [7]. Furthermore, structural mutations to growth factor receptors can lead to increased activation and sustained proliferative signalling even in the absence of ligand. Deregulation of the signal transduction cascades that follow growth factor receptor activation also contributes to sustained proliferative signalling. Deregulation of SOS-Ras-Raf-MAPK signalling is a common source of growth signal autonomy in cancer, and approximately a quarter of cancerous tumors possess a mutated constitutively active form of Ras, leading to hyperactivation of MAPK signalling and sustained growth [8]. Similarly, activation of the phosphoinositide-3-kinase (PI3K)/Akt/mTOR pathway leads to increased protein synthesis and sustained growth and survival [9].

Cancer cells also have an ability to ignore antigrowth signals. In normal tissues, quiescence and homeostasis are maintained through antiproliferative signalling, which can involve soluble growth inhibitors, extracellular matrix proteins, and surface proteins on nearby cells [3]. Many antigrowth signals are controlled by tumor suppressing retinoblastoma protein (pRb). When dephosphorylated, pRb sequesters and alters the function of E2F transcription factors, which govern the expression of genes necessary for progression from G1 into S phase. Antigrowth factors such as TGFβ block the phosphorylation of pRb and prevent cell cycle progression [10]. Cancer cells frequently have defects in the pRb pathway such as pRb loss of function by viral oncoproteins or mutation [11,12], downregulation or mutation of the TGFβ receptor [13,14], and defects in molecules such as Smad4 that transduce growth-supressing signals [15].

Another gene commonly mutated in cancer is *TP53*, which encodes the tumor suppressor p53. Nicknamed the ‘guardian of the genome’, p53 prevents tumor formation by halting the division of cells with mutated DNA to give time for repair and by initiating apoptosis of any cells mutated beyond repair through transcriptional regulation [16]. Under normal physiological conditions, p53 is a predominately nuclear transcription factor maintained at low levels by MDM2, an E3 ubiquitin ligase that binds p53, blocking its transcriptional activity and targeting it for ubiquitination, nuclear export, and proteasomal degradation [17]. A wide range of stressors, including hypoxia, oxidative stress, DNA damage, and nutrient deprivation, cause release of p53 from MDM2, leading to its activation and resulting in downstream effects such as autophagy, cell cycle arrest, DNA repair, and apoptosis, culminating in tumor suppression [16]. The function of p53 is frequently dysregulated in cancer, with *TP53* loss-of-function mutations occurring in approximately half of all human cancers [17,18]. Additionally, around 7% of tumors have amplification of the *mdm2* gene, leading to increased suppression of p53 [19].

Phosphatase and tensin homologue (PTEN) is a tumor-supressing phosphatase involved in cell metabolism, cell cycle regulation, cell motility and polarity, genomic maintenance, cell proliferation and survival, and senescence [20]. As a lipid phosphatase, PTEN negatively regulates Akt signalling by catalyzing the dephosphorylation of the 3′ phosphate of the inositol ring of PIP_3_ [21].

### 1.2. Reactive Oxygen Species (ROS)

Free radicals are atoms or molecules with one or more unpaired electrons in their orbitals. Oxidative stress occurs when there is an imbalance between the amount of ROS generated by a cell and the cell’s ability to scavenge and neutralize ROS or to repair damage caused by ROS and reactive intermediates. Increased oxidative stress has been implicated as a component of many pathologies, including cardiovascular disease [22], depression [23], Alzheimer’s disease [24], Parkinson’s disease [25], and cancer [26].

Intracellular ROS are generated in all cells as a by-product of several biological processes, including oxidative phosphorylation in the mitochondria, β-oxidation of fatty-acids in mitochondria and peroxisomes, and oxidation of proteins in the endoplasmic reticulum [27]. Enzymatic reactions involving cyclooxygenases, NADPH oxidases, lipoxygenases, xanthine oxidases, and the iron-catalyzed Fenton reaction are also sources of intracellular ROS. In addition to biological processes, stimuli such as radiation (nuclear, ultraviolet, and thermal) and chemotherapeutics can also increase intracellular ROS levels [27].

To protect against oxidative stress, cells have antioxidant defense systems that neutralize ROS and repair oxidative damage. Enzymatic antioxidants include superoxide dismutase (SOD) to convert superoxide into hydrogen peroxide, catalase to breakdown hydrogen peroxide into water and oxygen, and glutathione peroxidase (GPx) which uses glutathione (GSH) as a cofactor to reduce lipid peroxides and hydrogen peroxide [28]. Additionally, cells have non-enzymatic antioxidants which scavenge free radicals, including glutathione, vitamins E and C, and thioredoxin. These systems can be transcriptionally regulated by a variety of proteins, including activator protein-1 (AP-1), p53, forkhead box O (FOXO), hypoxia-inducible factor 1-α (HIF-1α), and NFκB, depending on the cell type and stimulus. The master regulator of the antioxidant response is the nuclear factor (erythroid-derived 2)-like 2 (Nrf2) transcription factor [29]. Under basal conditions, Nrf2 is bound to its inhibitor KEAP1, which targets it for ubiquitination and proteasomal degradation. ROS modify cysteine residues on KEAP1, resulting in a conformational change releasing Nrf2, allowing it to translocate to the nucleus where it binds to antioxidant response elements (AREs) within the promoter regions of genes for ROS detoxification, glutathione synthesis, NADPH regeneration, and drug metabolism [29].

ROS are implicated in the pathophysiology of cancer and may act as tumor promoters or suppressors depending on concentration, duration of exposure, and cellular context [30]. Moderate levels of ROS induce DNA damage, leading to genomic instability and activation of pro-survival signalling pathways, including PI3K/Akt, MAPK, and NFκB. This contributes to several hallmarks of cancer, including proliferation, evasion of apoptosis, and angiogenesis. However, when ROS levels exceed the antioxidant capacity of the cell, they cause oxidative damage to proteins, lipids, and nucleic acids, which can trigger apoptosis [31]. Oxidative damage to proteins disrupts their folding, leading to accumulation of misfolded proteins in the ER and activation of the unfolded protein response (UPR). This in turn can increase mitochondrial ROS production, creating a cycle of oxidative and ER stress [32,33]. Due to their altered metabolism and mitochondrial dysfunction, cancer cells have higher basal ROS levels than healthy cells, and as such, they may be particularly vulnerable to further ROS elevation [34].

### 1.3. Apoptosis Signal-Regulating Kinase 1 (ASK1)

**Figure 1:**
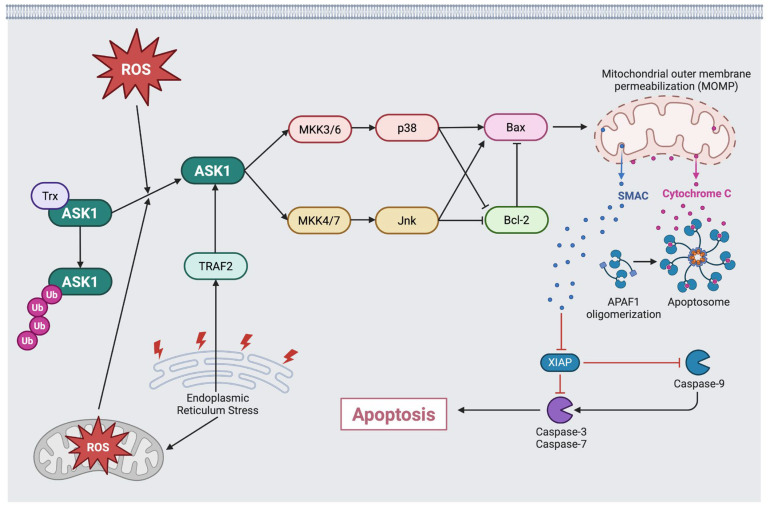
Activation and downstream signalling of apoptosis signal-regulating kinase 1 (ASK1). Under basal conditions, Trx inhibits ASK1 and targets it for ubiquitination and proteasomal degradation. Elevated levels of ROS oxidize Trx, causing it to dissociate from ASK1. This allows for autophosphorylation and activation of ASK1, leading to downstream activation of JNK and p38. ASK1 activation is also triggered by TRAF2 in response to ER stress. JNK and p38 activate proapoptotic Bax and inhibit antiapoptotic Bcl-2, causing an increase mitochondrial outer membrane permeabilization (MOMP) and release of apoptogenic factors from the mitochondria, which initiates apoptosis.

Apoptosis signal-regulating kinase 1 (ASK1) is a 1374-amino-acid protein consisting of an N-terminal thioredoxin (TRX)-binding domain, a central regulatory region, which contains a tumor necrosis factor receptor-associated factor (TRAF)-binding region, a serine/threonine kinase domain, and a coiled-coil region located at the C-terminus [35]. During stress-free conditions, ASK1 is inactive and exists in a complex known as the ‘ASK1 signalosome’ [36]. Within this complex, TRX binds the N-terminus of ASK1, targeting it for ubiquitination and proteasomal degradation [37], and 14-3-3 protein recognizes and binds pS^967^ to sequester and inhibit ASK1 [38,39,40,41]. During conditions of oxidative stress, TRX dissociates from ASK1 [42] and S^967^ is dephosphorylated, leading to the dissociation of 14-3-3 [43], ultimately allowing for the recruitment of TRAF2 and TRAF6 by the central regulatory region of ASK1 [36]. This in turn promotes homo-oligomerization of ASK1 at the N-terminus, which induces an open conformation of the central regulatory region and autophosphorylation of the activation loop, thereby activating ASK1 and enabling its association with the downstream MAP2K [35,36,44,45]. Specifically, ASK1 phosphorylates and activates MKK4/7 and MKK3/6, which in turn activate JNK and p38, respectively. JNK and p38 in turn phosphorylate pro-apoptotic proteins like bim and bax, causing their translocation to the mitochondria and inducing the release of apoptogenic factors. JNK and p38 also inactivate pro-survival proteins such as Bcl-2 and Bcl-xL (Figure 1).

ASK1 is also activated independently of oxidative stress through the unfolded protein response (UPR). Under conditions of ER stress, accumulation of misfolded proteins in the ER displaces the chaperone binding immunoglobulin protein (BiP/GRP78) from IRE1α, PERK, and ATF6, leading to their activation [33]. IRE1α recruits TRAF2, which associates with ASK1 to form an IRE1α-TRAF2-ASK1 complex, driving downstream JNK activation and apoptosis [33,46]. PERK leads to downstream induction of the transcription factor C/EBP homologous protein (CHOP), which promotes pro-apoptotic gene expression. ER stress also potentiates ASK1 activation directly through Ca^2+^ signalling [47] and indirectly through mitochondrial ROS generation [32]. Physical contacts between the ER and mitochondria at mitochondria-associated membranes (MAMs) facilitate calcium transfer from the ER to the mitochondria during ER stress, disrupting electron transport chain function and increasing mitochondrial ROS production [48]. This ROS can in turn activate ASK1 via Trx dissociation. Ca^2+^ signalling also activates ASK1 through Ca^2+^/calmodulin-dependent protein kinase type II (CaMKII), which directly phosphorylates ASK1 at the T^845^ (T^838^ in humans) residue [47,49,50].

ASK1 promotes apoptosis under stress conditions and may be a potential target for cancer treatment. In gastric cancer patients, ASK1 mRNA levels were lower in patients with lymph node metastases compared to those with no lymph node metastases [51]. Furthermore, lower levels of ASK1 mRNA were found in HGC-27 gastric cancer cells compared to non-cancerous GES-1 gastric epithelial cells, and increasing ASK1 expression in HGC-27 cells using lentiviral transduction resulted in inhibition of proliferation and migration and activation of JNK and p38, suggesting that ASK1 may be a potential therapeutic target to inhibit proliferation and invasion of gastric cancer [51].

Overexpression of wildtype ASK1 in NSCLC cells (A549 and H1975) resulted in decreased proliferation and migration, but overexpression of kinase-dead mutant ASK1 had no inhibitory effects on proliferation or migration [52]. Additionally, ASK1 was found to interact with YAP and TAZ by binding through their WW domains, and ASK1 overexpression downregulated transcription of the YAP/TAZ target genes *CYR61* and *CTGF* and downregulated protein levels of CYR61. Furthermore, ASK1 overexpression blocked nuclear localization of TAZ, and knockdown of TAZ inhibited proliferation and migration to a similar extent as ASK1 overexpression [52]. Glutathione peroxidase-1 (GPx1), an enzyme involved in protecting against oxidative stress, was found to negatively regulate TNF-α-induced apoptosis in RIPK3-negative cancer cells via two mechanisms involving suppression of ASK1 [53]. Firstly, GPx1 reduces hydrogen peroxide, which keeps Trx in the reduced state, where it is bound to and inhibits ASK1 [53]. Additionally, GPx1 interferes with formation of the active ASK1 complex by binding the zinc finger domain of TRAF2, which prevents association of TRAF2 with ASK1 and decreases the local concentration of hydrogen peroxide, which keeps Trx bound to ASK1 [53]. Overall, these data indicate that ASK1 may be a therapeutic target to inhibit cancer cell proliferation and migration by inactivating TAZ.

While many studies support the anticancer potential of ASK1, it is important to note that ASK1 may be pro-survival and tumorigenic in some contexts. ASK1 is overexpressed in human pancreatic cancer specimens and cell lines, and its expression correlates with the histological grade of the tumor [54]. Knockdown of ASK1 in PANC1 cells impaired proliferation and colony formation and impaired tumor formation in a xenograft model [54]. Human gastric cancer specimens were also found to have increased expression of ASK1 relative to non-tumor epithelium, and ASK1 knockdown inhibited chemically induced gastric tumorigenesis in a mouse model [55]. ASK1 was also shown to facilitate cancer metastasis, and ASK1 deletion in platelets attenuated metastasis in mice [56].

## 2. Evidence of ASK1 as an Anticancer Target

There is a growing body of evidence that supports the anticancer potential of activation of ROS/ASK1 signalling. Here, we present a narrative review outlining several preclinical studies across lung, breast and gynecologic, and gastrointestinal cancer models that demonstrate anticancer effects with ASK1 involvement. Findings are presented in order from weakest to strongest evidence implicating ASK1 in the anticancer mechanism. Studies that only show an association between anticancer effects and ASK1 activation were considered the weakest tier of evidence. The next-higher-evidence tier were studies where up- and downstream nodes of ASK1 signalling were perturbed (i.e., ROS or MAPK inhibition, but not ASK1 inhibition). The next tier of evidence were studies that used direct pharmacological inhibition of ASK1. The highest tier of evidence were studies that used direct knockdown of the ASK1 protein.

### 2.1. ASK1 Mediates Anticancer Effects in Lung Cancer and Mesothelioma In Vitro

Treatment of A549 NSCLC cells with silver nanoparticles (1–50 µg/mL) resulted in significantly decreased cell viability and increased apoptosis after 5 h [57] (Table 1). This corresponded with increased levels of intracellular ROS, c-casp-3, and phosphorylated ASK1, JNK, and p38. Overall, these data suggest that silver nanoparticles induce apoptosis of A549 cells through a mechanism which may involve ROS and activation of ASK1. This study could be improved by checking whether silver nanoparticles still produce anticancer effects if ASK1 activation is blocked.

Treatment of H1299 NSCLC cells with indole-3-carbinol (100–700 µM), a phytochemical abundant in cruciferous vegetables, for 24 and 48 h resulted in decreased proliferation, decreased colony formation, increased apoptosis, increased sub-G1 cell cycle arrest, and increased DNA fragmentation [58] (Table 1). These anticancer effects of indole-3-carbinol were accompanied by a robust increase in levels of ROS, and NAC (5 mM) significantly attenuated the indole-3-carbinol-induced decrease in cell viability and apoptosis. Indole-3-carbinol treatment increased levels of c-PARP, c-casp-3, c-casp-7, and c-casp-9—modulators of intrinsic apoptosis [58]. Additionally, indole-3-carbinol increased levels of γ-H2AX, FOXO3, Bax, and Bim and decreased levels of pAkt, Bcl-2, and Bcl-xL, further proving that indole-3-carbinol induces apoptosis of H1299 cells. Indole-3-carbinol also increased levels p-ASK1, decreased levels of Trx1 and Prdx-1, and NAC attenuated these effects, suggesting that the indole-3-carbinol-induced apoptosis of H1299 cells is mediated by increased ROS and ASK1 activation [58].

Treatment of A549 and PC9 NSCLC cells with the isoquinoline alkaloid berberine (BBR) resulted in concentration- and time-dependent inhibition of proliferation and induction of apoptosis, as indicated by flow cytometry and increased levels of cleaved caspase-3 and cytosolic cytochrome C and an increased Bax/Bcl-2 ratio [59]. BBR (40–80 µM) increased ASK1 phosphorylation (Thr^845^) and phosphorylation of JNK. The JNK inhibitor SP600125 partially attenuated BBR-induced apoptosis. BBR was also found to increase intracellular ROS levels, and pretreatment with the ROS scavenger NAC blocked the increased phosphorylation of ASK1 and JNK and attenuated BBR-induced apoptosis. Taken together, these data suggest that the anticancer effects of BBR are mediated by induction of ROS and activation of ASK1 signalling [59].

Zhang et al. [60] investigated the effects of (E)-2-(2-chlorostyryl)-3,5,6-trimethylpyrazine (CSTMP)—a synthetic compound that combines pharmacophores from resveratrol and tetramethylpyrazine (TMP)—in A549 NSCLC cells. CSTMP treatment (50–150 µM; 24–96 h) caused concentration- and time-dependent inhibition of cell viability, G0/G1 cell cycle arrest, induction of apoptosis, and induction of ER stress. Apoptosis was indicated by annexin-V/PI staining, increased levels of cleaved PARP and cleaved caspases-3, -8, and -9, and an increased Bax/Bcl-2 mRNA ratio. Endoplasmic reticulum stress was evidenced by increased mRNA levels of GRP78, GRP94, and CHOP and increased cleavage of the ER-stress-activated caspases-4 and -12 [60]. CSTMP upregulated IRE1α, TRAF2, p-ASK1, and p-JNK, and immunoprecipitation using IRE1α antibody showed that CSTMP induced association of ASK1 and TRAF2 with IRE1α. IRE1α knockdown using siRNA attenuated the effects of CSTMP. Taken together, these data show that pharmacologic induction of ER stress can activate an IRE1α-TRAF2-ASK1 complex, leading to JNK activation and caspase-dependent apoptosis [60]. While this study does not directly implicate ASK1, it does link ER stress to ASK1 activation through IRE1α.

Treatment of A549 and H1975 NSCLC cells with a combination of daidzein (300 μM), a soy-derived isoflavone phytochemical, and gefitinib (10 μM), a first-generation EGFR tyrosine kinase inhibitor, resulted in synergistic inhibition of cell viability and induction of apoptosis with increased levels of Bid, Bax, and Bad, cytochrome c, FasL, FAS, and FADD, c-caspases-8, -3, -9, and c-PARP, and decreased levels of Bcl-2 and Bcl-xL [61]. Combination treatment significantly increased both mitochondrial and cellular ROS levels, as demonstrated by MitoSOX Red and H2DCFDA staining. Pretreatment with the ROS scavenger NAC attenuated the decreased cell viability and induction of apoptosis [61]. Combination treatment increased levels of p-ASK1 and p-JNK and increased nuclear localization of c-Jun. Additionally, the combination suppressed the EGFR/STAT/AKT/ERK survival pathway through inhibition of multiple EGFR phosphorylation sites and downstream effectors while upregulating PTEN and downregulating the drug resistance transporter ABCG2 [61]. Combination treatment also induced G0/G1 cell cycle arrest with increased expression of p53, p27, p21, and p16 and decreased expression of cyclin D1 and cyclin E. Collectively, these data demonstrate that daidzein synergizes with gefitinib to enhance lung cancer cell death via ROS-mediated ASK1/JNK/c-Jun signalling activation, suppressing pro-survival EGFR pathways, and inducing cell cycle arrest and apoptosis.

Treatment of H460 and H1975 NSCLC cells with ecliptasaponin A, a phytochemical purified from *Eclipta prostrata*, inhibited cell viability and colony formation and induced apoptosis [62] (Table 1). These results corresponded with increased levels of cleaved caspases-3, -8, and -9, and pre-treatment with the pan-caspase inhibitor z-VAD (50 µM) attenuated ecliptasaponin-A-induced apoptosis. Additionally, ecliptasaponin A induced autophagy, as indicated by increased expression of LC3, Beclin-1, and p62, and co-treatment with autophagy inhibitors (5 mM 3-MA or 20 µM CQ) decreased the proportion of apoptotic cells and blocked the ecliptasaponin-A-induced increase in c-casp-3 [62]. Furthermore, ecliptasaponin A increased phosphorylated levels of ASK1, JNK, and Akt (Ser^473^ and Thr^308^) and decreased phosphorylation levels of Erk. Blocking ASK1 activation using GS-4997 (1 µM) or JNK activation using SP600125 (20 µM) attenuated ecliptasaponin-A-induced apoptosis and autophagy.

Denbinobin (20 µM), a phytochemical isolated from plants of the genus *Dendrobium*, induced apoptosis of wildtype A549 NSCLC cells and increased activity of ASK1, but this effect was attenuated by an ASK1 dominant-negative (ASK1DN) mutant phenotype [63] (Table 1). Additionally, denbinobin increased ROS levels in A549 cells, and pre-treatment with the antioxidants NAC (1 mM) or GSH (100 µM) markedly attenuated the denbinobin-induced increase in ASK1 activity and apoptosis of A549 cells, indicating that the effects are ROS-dependent [63]. Denbinobin caused a time-dependent increase in phosphorylation and activity of JNK, and pre-treatment with the JNK inhibitor SP600125 (10 µM) attenuated denbinobin-induced apoptosis. Furthermore, NAC, GSH, and ASK1DN significantly attenuated denbinobin-induced JNK phosphorylation. Denbinobin increased AP-1 protein complex formation, and pre-treatment with curcumin (1 µM), an AP-1 inhibitor, attenuated denbinobin-induced apoptosis [63]. Denbinobin increased p-c-Jun, and this was significantly attenuated by NAC, GSH, SP600125 (JNK inhibitor), ASK1DN, and JNK1/2 dominant-negative (JNK1/2DN). Denbinobin increased Bim mRNA levels, and transfection with Bim-siRNA attenuated denbinobin-induced apoptosis. Moreover, NAC, GSH, SP600125 (JNK inhibitor) ASK1DN, and JNK1/2DN all blocked denbinobin-induced Bim mRNA expression. Overall, these data indicate that the proapoptotic effects of denbinobin rely on activation of an ROS-ASK1-JNK-c-Jun signalling cascade to induce AP-1 activation and Bim expression [63].

Treatment of H28 mesothelioma cells with diarachidonoylphosphoethanolamine (DAPE; 1–100 µM) caused concentration- and time (24–48 h)-dependent inhibition of cell viability and induction of apoptosis independent of caspase activation [64] (Table 1). DAPE increased ROS levels in H28 cells, and DAPE-induced inhibition of cell viability and induction of ROS was blocked by the antioxidants NAC (1 mM) and glutathione (1 mM), the NOX inhibitor Vas-2870 (1 µM), and the Rac inhibitor NSC23766 (10 µM), suggesting that DAPE stimulates NOX-dependent ROS generation to promote apoptosis. DAPE decreased thioredoxin reductase (TrxR) activity, induced dissociation of Trx from ASK1, and increased phosphorylation (Thr^845^) of ASK1, increased phosphorylation (Thr^180^/Tyr^182^) of p38, and increased phosphorylation (Thr^183^/Tyr^185^) of JNK and glutathione prevented the increased phosphorylation of ASK1, p38, and JNK [64]. Additionally, DAPE-induced p38 activation was blocked by siRNA knockdown of ASK1, although ASK1-siRNA did not block DAPE-induced JNK activation. Overall, these results indicate that DAPE promotes caspase-independent apoptosis of H28 cells by stimulating NOX-dependent ROS production, inhibiting TrxR, and activating ASK1/p38 signalling [64].

### 2.2. ASK1 Mediates Anticancer Effects in Breast and Gynecologic Cancer In Vitro

Treatment of breast cancer cells, (MCF-7, MDA-MB-468 and SK-BR-3) with cambogin—a compound isolated from the genus *Garcinia*, significantly inhibited proliferation and induced apoptosis with a concomitant increase in the Bax/Bcl-2 ratio [65]. The proapoptotic effect of cambogin was attenuated by Bcl-2 overexpression and by siRNA knockdown of Bax and/or Bak. Cambogin had no effect on expression of caspases-3, -9, and –8, suggesting that its effect on apoptosis is caspase-independent. This was confirmed as the caspase inhibitor Z-VAD-FMK did not rescue cells from cambogin-induced cell death. Furthermore, western blotting and immunofluorescence staining showed that the treatment caused nuclear translocation of AIF, consistent with caspase-independent apoptosis. Proteomics analysis showed that 101 proteins that were either up- or downregulated, 10 of which are directly involved in production and accumulation of reactive oxygen species, and 43 are indirectly involved, suggesting ROS as a potential mechanism of cambogin-induced apoptosis. This was confirmed as co-treatment with the ROS scavenger NAC blocked the cambogin-induced increase in the Bax/Bcl-2 ratio and nuclear translocation of AIF. Most importantly, cambogin treatment increased phosphorylation of ASK-1, MKK4 and MKK7. Together, these results indicate that the proapoptotic effect of cambogin in breast cancer cells is mediated by ROS and ASK1 activation.

Treatment of MDA-MB-231 and MDA-MB-468 breast adenocarcinoma cells with 0.2–0.8 µM pristimerin for 24–72 h resulted in concentration- and time-dependent inhibition of cell viability with minimal effect on the viability of MCF-10A immortalized breast epithelial cells [66] (Table 2). Pristimerin induced G1-phase cell cycle arrest in breast cancer cells, as indicated by flow cytometry, decreased levels of cyclin D1 and CDK4, and increased levels of p53 and p21. Pristimerin induced apoptosis, as indicated by flow cytometry, nuclear fragmentation, cell shrinkage, and increased levels of c-caspase-3 and c-PARP, and co-treatment with the pan-caspase inhibitor z-VAD partially restored cell viability. Pristimerin induced autophagy, as indicated by accumulation of acidic vesicles (AO/EB staining) and increased expression of LC3-II, beclin-1, and p62, and inhibition of autophagy using 3-methyladenine partially attenuated the pristimerin-induced decrease in cell viability. Blocking apoptosis using z-VAD also blocked pristimerin-induced autophagy, and blocking autophagy using 3-methyadenine also blocked pristimerin-induced apoptosis [66]. Treatment with pristimerin increased phosphorylated levels of JNK and p38, and inhibition of JNK using SP600125 partially attenuated the effects of pristimerin on cell viability. Pristimerin treatment caused a potent increase in levels of intracellular ROS, and pre-treatment with NAC completely blocked pristimerin-induced inhibition of viability, induction of apoptosis, and induction of autophagy [66]. Pristimerin caused concentration-dependent inhibition in Trx-1 activity and an increase in phosphorylated (Thr^845^) levels of ASK1, and pre-treatment with NAC blocked these effects.

Treatment of BGC-823 cells (a HeLa-derived cell line that is misidentified as human gastric cancer) with 5–40 µM curcumin for 24 h caused a concentration-dependent reduction in cell viability, an increase in intracellular ROS levels, and a decrease in GSH/GSSG ratio [67]. Treatment with 20 µM curcumin for 24 h induced apoptosis (annexin V/PI and caspase-3 activity), and this effect was attenuated when cells were pretreated with NAC—an ROS scavenger, Tiron—a vitamin E analogue, or SP600125—a JNK inhibitor. The effects of curcumin were associated with increased levels of p-ASK1 (Thr^845^), p-MKK4 (Thr^261^), and p-JNK (Thr^183^/Tyr^185^), but pre-treatment with NAC prevented the phosphorylation of these proteins [67]. Overall, these results indicate that the anticancer properties of curcumin may be attributable to induction of ROS and subsequent activation of an ASK1-MKK4-JNK signalling cascade to trigger apoptosis.

Scaffolding protein claudin 6 (CLDN6) has breast cancer suppressor gene properties and is downregulated in MCF-7 breast cancer cells [68]. Overexpressing CLDN6 in MCF-7 cells increased activation of ASK1 through decreased inhibitory phosphorylation at S^967^ residue, with a corresponding increase in phosphorylation of JNK and p38. Protein expression of RIP1 was also increased. These effects of CLDN6 overexpression were reversed by treatment with the ASK1 inhibitor TRX1 (10 ng/mL, 48 h), which increased p-ASK1 (S^967^) and decreased p-JNK and p-p38. Transfected cells had an increase in apoptosis, but this was attenuated by TRX1, further supporting that the anticancer effect of CLDN6 overexpression in MCF7 cells is mediated through ASK1 signalling. Rho-123 fluorescence staining indicated that TRX1 treatment reduced mitochondria membrane potential in transfected cells, and mitochondria structure analysis confirmed that treatment reduced disruption, which indicated that CLDN6 induced apoptosis via activation of the mitochondrial apoptosis pathway. Transfected MCF-7 cells showed significantly decreased Bcl-2, and significantly increased Bax protein expressions as compared to the non-transfected controls, and this was reversed when treated with TRX1. Finally, while MCF-7 cells do not normally express caspase-3, transfection with CLDN6 has been shown to induce expression of caspase-3 protein. In this study, caspase-3 was present and activated in the transfected cells, and this was inhibited by TRX1. Taken together, these data demonstrate that CLDN6 is a tumor suppressor, which inhibits MCF-7 cell survival and induces apoptosis through activation of ASK1, a decreased Bcl-2/Bax ratio and increased caspase-3 activation.

Inhibition of pyruvate dehydrogenase kinase isozyme 4 (PDK4) in breast cancer cells activated the ASK1/JNK MAP kinase signalling pathway, leading to increased autophagy-dependent ferroptosis [69]. PDK4 is overexpressed in breast cancer cells (MDA-MB-231, MCF-7 and SUM190PT) as compared to non-tumorigenic mammary cells (MCF-10A). Increased PDK4 is associated with poor patient outcome; therefore, Shi et al. [69] used siRNA to knockdown expression and examine the role of increased PDK4 in autophagy. In MCF-7 cells, the downregulation of PDK4 induced an increase in phosphorylation of ASK1 and JNK, suggesting PDK4 silencing is an activator of ASK1/JNK signalling in breast cancer. Transfected si-PDK4 MCF-7 cells were treated with the ASK1 inhibitor GS-4997, leading to decreased phosphorylated ASK1 and decreased phosphorylated JNK. Knockdown of PDK4 increased lipid production and intracellular ROS, which was abolished with the ASK1 inhibitor. This suggests that PDK4 inhibition in breast cancer cells causes activation of ASK1/JNK signalling and the promotion of autophagy-dependent ferroptosis.

Treatment of HeLa human cervical adenocarcinoma and SiHa human cervical squamous cell carcinoma cells with 10–50 µM α-mangostin for 24–48 h caused concentration-dependent induction of apoptosis and inhibition of cell viability [70] (Table 2). These results coincided with a concentration-dependent increase in levels of cleaved caspase-3, cleaved caspase-9, and cleaved PARP, and pre-treatment with a pan-caspase inhibitor (Z-VAD) attenuated α-mangostin-induced cell death. Furthermore, α-mangostin treatment caused a concentration-dependent reduction in mitochondrial membrane potential with a corresponding increase in levels of the pro-apoptotic proteins Bax and cytochrome c and a decrease in levels of the pro-survival protein Bcl-2 [70]. Levels of p-p38 and ROS were elevated following α-mangostin treatment, and inhibition of p38 using SB203580 or siRNA or inhibition of ROS using NAC attenuated the pro-apoptotic effect of α-mangostin. Additionally, α-mangostin treatment caused a concentration-dependent increase in levels of p-ASK1 and p-MKK3/6, and siRNA knockdown of either of these proteins attenuated the anticancer effects of α-mangostin [70]. Overall, these results indicate that the pro-apoptotic and antitumor effects of α-mangostin involve induction of intracellular ROS and subsequent activation of ASK1, MKK3/6, and p38.

Treatment of ovarian cancer cells with metformin (2–16 mM) under low-glucose conditions (0–2.5 mM) enhanced metformin-induced inhibition of cell viability and induction of apoptosis, as indicated by increased levels of Bax, cytochrome c, and c-casp-3, decreased levels of Bcl-2, and decreased mitochondrial membrane potential [71] (Table 2). Interestingly, under low-glucose conditions, metformin increased levels of noxa—a pro-apoptotic protein associated with mitochondrial damage—increased levels of p-ASK1 and p-JNK, increased levels of Grp78 and CHOP (markers of ER stress), and increased levels of c-casp-4—a marker of ER stress-induced apoptosis. Pre-treatment with NQDI-1, a pharmacological ASK1 inhibitor, restored Noxa and Bcl-2 levels to basal and blocked low-glucose metformin-induced increase in p-JNK, CHOP, and c-casp-4 [71]. ASK1-siRNA abolished both the metformin plus low glucose-induced increase in number of apoptotic cells and the reduction in mitochondrial membrane potential. The effects of metformin under low-glucose conditions corresponded with increased ROS levels, and inhibition of ROS using NAC attenuated many of the effects of low glucose combined with metformin, including increased p-ASK1, increased noxa, decreased mitochondrial membrane potential, increased casp-3 activity, and increased c-casp-4 levels. Additionally, the ER stress inhibitor TUDC inhibited low-glucose metformin-induced apoptosis [71].

### 2.3. ASK1 Mediates Anticancer Effects in Gastrointestinal Cancer In Vitro

Treatment of SNU-213 human pancreatic adenocarcinoma cells with 200–600 µM naringenin for 24 h caused a dose-dependent reduction in cell viability and an increase in intracellular ROS and apoptosis [72]. Naringenin upregulated levels of pro-apoptotic Bak and cytochrome c and decreased levels of pro-survival Bcl-XL. These results coincided with increased levels of cleaved PARP and cleaved caspases-3, -7, and -9. Additionally, naringenin treatment decreased levels of Prdx-1 and increased levels of ASK1, p-JNK, p-p38, and total and p-p53 [72]. Overall, these data indicate that the proapoptotic effects of naringenin in pancreatic cancer cells may be mediated by increased intracellular ROS, inhibition of Prdx-1, and activation of ASK1 signalling.

Pancreatic cancer cell lines SW1990 and L3.7 had reduced proliferation in a dose-dependent manner when treated with Erianin for 48 h, with IC_50_ values of 472.8 and 101.0 nM, respectively [73]. Erianin inhibits the progression of pancreatic cancer by directly targeting AKT and ASK1. A colony formation assay showed significantly reduced viability and significantly reduced clones following treatment. Erianin treatment decreased the migratory abilities of these pancreatic cancer cells seen in both wound healing assays and transwell assays. Western blot analysis showed decreased N-cadherin, vimentin and b-catenin, while E-cadherin was increased, which confirms the decrease in migratory characteristics. Erianin treatment interrupted the cell cycle, which was shown by flow cytometry analysis revealing cells arrested in G2/M phase. Furthermore, erianin significantly increased the rate of apoptotic cells and the production of reactive oxygen species. Erianin treatment inhibited AKT/FOXO signalling while activating ASK1/JNK/p38 MAPK pathways, as evidenced by RNA sequencing and upregulation of phosphorylation seen with Western blotting. Finally, molecular docking studies showed that erianin has binding potential to ASK1 and AKT directly.

In HT-29/NC colon adenocarcinoma cells, with high nicotinamide N-methyltransferase (NNMT), a metabolic enzyme upregulated in various tumors, and in HT-29/shNNMT colon cancer cells, with low expression of NNMT, 2.5–4 mM vanillin (Van) treatment for 48 h decreased mRNA and protein levels of NNMT [74]. Treatment of HT-29/NC, HT-29/shNNMT, and SW480/NC and SW480/NNMT, NNMT-overexpressing, cells, with Van decreased cell viability [74]. Furthermore, treatment of HT-29/NC, HT-29/shNNMT, SW480/NC, and SW480/NNMT cells with Van decreased colony formation and increased apoptosis, with a greater effect in HT-29/NC and SW480/NNMT cells, which express increased NNMT [74]. Additionally, Van treatment increased p53 and cleaved PARP, caspase-3, and caspase-9 protein levels in all cell lines [74]. Furthermore, fluorouracil (5-Fu), a chemotherapeutic, in combination with Van increased apoptosis in all cell lines greater than either treatment alone, with greater effects in HT-29/NC and SW480/NNMT cell lines [74]. Additionally, Van treatment increased p-p38 and p-ASK1 protein levels; however, in the presence of SB203580, a p38 inhibitor, Van induced an increase in p-p38 proteins levels, and apoptosis was attenuated in all cell lines [74]. Van treatment increased intracellular ROS and decreased MMP, as evidenced by JC-1 staining. The ROS inhibitor NAC attenuated the effects of Van in all cell lines [74]. These data indicate that Van treatment induced apoptosis through inhibition of NNMT and activation of ROS/ASK1/p38 signalling.

ST13 is a cofactor of heat shock protein, and its expression is downregulated in colorectal and breast cancer [75,76]. Overexpression of ST13 in HCT116 colorectal carcinoma cells using adenoviral transduction reduced cell viability, increased transcriptional activity of AP-1, and induced apoptosis, as indicated by flow cytometry, increased levels of cleaved caspase-3, and increased cytosolic levels of cytochrome c [77] (Table 3). These results coincided with increased expression of p-ASK1, p-JNK, and p-c-Jun, and siRNA knockdown of ASK1 or JNK restored cell viability and inhibited apoptosis of ST13-overexpressing cells [77]. Overall, these results indicate that the tumor supressing properties of ST13 are mediated by increased activation of ASK1 and JNK signalling.

Atmospheric pressure gas plasma (AGP) treatment of colorectal cancer cell lines (Caco2, HCT116, and SW480) for 5, 15, and 30 s increased intracellular ROS, decreased cell viability, and increased caspase-3/-7 activation, with no effect in normal control cells (CO18, MRC5, and FHC) [78]. Importantly, AGP treatment significantly increased phosphorylation of ASK1 and p38, a key MAPK downstream of ASK1 [78]. In the presence of NAC, an ROS scavenger, the increase in ASK1 and p38 phosphorylation levels was attenuated [78]. Additionally, siRNA knockdown of ASK1 decreased AGP-induced p38 phosphorylation, increased cell viability, and decreased caspase-3/-7 activity and ROS production, indicating the importance of ASK1 in AGP treatment of colorectal cancer cell lines [78].

Treatment of colorectal cancer cells (HT29, SW480, and LoVo) with cisplatin (1–30 µM) for 48 h caused concentration-dependent inhibition of cell viability [79] (Table 3). The colorectal cancer cells were found to have increased basal expression of miR-20a compared to FHC normal colon epithelial cells, and further increasing miR-20a levels decreased sensitivity to cisplatin, while knocking down miR-20a by transfection with anti-miR-20a enhanced sensitivity to cisplatin. Both cisplatin alone and cisplatin anti-miR-20a co-treatment increased intracellular ROS levels, and pre-treatment with NAC significantly attenuated the cytotoxicity of cotreatment. In silico analysis showed that the 3′ UTR of ASK1 mRNA has a complementary sequence to miR-20a, suggesting that ASK1 is a likely target of miR-20a, and this was confirmed using a dual-luciferase reporter assay [79]. Additionally, overexpression of miR-20a decreased the expression of ASK1, while anti-miR-20a increased total ASK1 expression and enhanced cisplatin-dependent ASK1 phosphorylation, and pre-treatment with NAC blocked ASK1 phosphorylation by cisplatin and anti-miR-20a cotreatment. Anti-miR-20a enhanced cisplatin-dependent phosphorylation of JNK, and this effect was blocked by siRNA knockdown of ASK1, whereas ASK1 overexpression further enhanced cisplatin-dependent phosphorylation of JNK [79]. Pre-treatments with NAC, siRNA-ASK1, or JNK inhibitor (SP600125) all attenuated the cytotoxicity of cotreatment with anti-miR-20a and cisplatin. Together, these data indicate that anti-miR-20a promotes cisplatin-induced toxicity via an ROS/ASK1/JNK signalling pathway. Cisplatin and anti-miR-20a cotreatment enhanced cisplatin-induced apoptosis, as indicated by flow cytometry, decreased mitochondrial membrane potential, increased phosphorylation/inhibition of the pro-survival protein Bcl-2, and increased levels of cytochrome c, smac/DIABLO, and cleaved caspases-3, -7, and -9. Disruption of ROS/ASK1/JNK signalling using NAC, siRNA-ASK1, or SP600125 attenuated the pro-apoptotic effect of cisplatin and anti-miR-20a cotreatment [79].

### 2.4. In Vivo Evidence of ASK1 as an Anticancer Target

Several in vivo studies support the role of ASK1 in cancer cell death (Table 4). Administration of ecliptasaponin A (25–50 mg/kg) to H460 xenografted mice resulted in decreased tumor size, indicating that the anticancer effects of ecliptasaponin are reproducible in vivo, although an in-depth investigation of the signalling mechanism was not performed [62]. Chen et al. [59] administered BBR (500 mg/kg) every other day to nude mice xenografted with A549 cells, and IHC analysis of tumors showed increased levels of p-ASK1 (Thr^845^), p-JNK, BAX, and c-casp-3; however, the study was only 30 days long, and the authors did not report tumor volume or survival data.

Stronger evidence of the anticancer potential of ASK1 is demonstrated by studies that showed associations between antitumor efficacy and ASK1 activation in lung, breast and gynecologic, and GI cancers. A549 xenografted nude mice administered a daidzein (300 mg/kg)/gefitinib (100 mg/kg) combination by oral gavage twice weekly for five weeks had improved survival and reduced tumor size, and this corresponded with increased TUNEL staining (confirming apoptosis) and increased levels of p-ASK1, p-JNK, and nuclear localization of c-Jun [61]. Taken together, these data support the in vitro findings that ASK1 activation has beneficial effects against lung cancer.

ASK1 activation was also associated with antitumor effects in breast and gynecologic cancer models. Intraperitoneal injection of α-mangostin (20–40 mg/kg, three times per week) in HeLa xenografted nude mice decreased the weight and volume of tumors and increased levels of p-ASK1, p-p38, c-caspase-3, and c-PARP [70]. Intraperitoneal injection of pristimerin (0.5 mg/kg, every other day) in MDA-MB-231 xenografted nude mice decreased the weight and volume of tumors, increased levels of p-ASK1 (Thr^845^), p-JNK, and LC3-II, and decreased the activity of Trx-1 [66].

Antitumor efficacy was also associated with ASK1 activation in gastrointestinal cancers. Nude mice xenografted with LoVo cells transfected with or without anti-miR-20a were administered 10 mg/kg cisplatin twice weekly for 28 days; anti-miR-20a rendered tumors more sensitive to cisplatin and tumor tissue had decreased levels of miR-20a and increased levels of phosphorylated ASK1 and JNK when compared to the tumors that did not have miR-20a knocked down [79]. Administration of erianin to SW1990 xenografted nude mice significantly inhibited tumor growth and increased phosphorylated levels of JNK and p38, cleaved-caspase-3, and decreased levels of ki-67 and phosphorylated Akt in tumor tissues [73].

While these studies point toward ASK1 as a potential mediator of anticancer effects, it should be noted that no ASK1 knockdown or pharmacological inhibitor experiments were conducted. In one study, administration of metformin (200–600 mg/kg/day) to SKOV3 xenografted mice resulted in a dose-dependent reduction in tumor volume and weight as well as increased levels of Grp78, CHOP, c-casp-3, and phosphorylated ASK1 [71]. Importantly, the antitumor effects of metformin were attenuated when administered alongside NQDI-1 (1 mg/kg/day), an ASK1 inhibitor directly implicating ASK1 in the antitumor effects of metformin [71].

## 3. Conclusions

Across lung, breast and gynecologic, and gastrointestinal cancers, ASK1 emerges as a potential mediator of apoptosis in response to a range of natural and synthetic compounds, especially those that trigger oxidative and endoplasmic reticulum stress. Despite variation in compound class, cancer type, and experimental model, the downstream consequences of ASK1 activation are conserved across many studies: phosphorylation and activation of MKK4/7 and MKK3/6, downstream JNK and p38 signalling, mitochondrial dysfunction, and caspase-dependent cell death. In vivo xenograft studies in multiple cancer models confirm an association between reduced tumor volume and ASK1 activation; however, additional studies using pharmacological or genetic ASK1 inhibition should be performed to establish ASK1 as mechanistically central.

While ASK1 activation is a consistent finding, the upstream mechanisms that trigger this activation are more difficult to disentangle. Two principal routes emerge from the reviewed literature: direct ROS generation leading to Trx oxidation and dissociation from ASK1 and ER-stress-mediated activation via the IRE1α-TRAF2-ASK1 complex. However, as discussed in the introduction, these pathways are deeply intertwined. Compounds may generate ROS directly or act on mitochondria to increase mitochondrial ROS, both of which would cause Trx oxidation and ASK1 activation. Additionally, increased ROS may also cause oxidative protein damage, ER stress, and UPR activation, which can in turn further generate mitochondrial ROS. This cycle of crosstalk between the ER and mitochondria means that ROS and ER stress may act in concert rather than independently, and the relative contribution of each pathway likely varies by compound, cancer type, and cellular redox context. Studies that rely solely on NAC pre-treatment to implicate ROS, or on GRP78/CHOP upregulation to implicate ER stress, may not fully capture the mechanistic complexity of this crosstalk.

Additionally, the limitations of the use of NAC as an ROS scavenger should be noted. Across many studies, NAC is framed as an ROS scavenger used to implicate ROS in the mechanism of action of different test compounds. In reality, NAC does a poor job directly scavenging physiologically relevant ROS such as peroxide and superoxide and acts through more indirect mechanisms to decrease intracellular ROS levels [80,81]. As a precursor of L-cysteine, NAC replenishes depleted glutathione (GSH)—a substrate for GPx which detoxifies peroxide and lipid hydroperoxides [80]. NAC also increases generation of hydrogen sulfide (H_2_S), which is converted to sulfane sulfur species, which are potent antioxidants [80]. NAC also inhibits transferrin-receptor-mediated iron uptake, which decreases the production of intracellular ROS by limiting iron-catalyzed Fenton reactions [81].

Despite the potential ASK1 holds as a therapeutic target, several challenges remain. Its context-dependent role in cell survival, limited translational data, and incomplete understanding of compound specificity warrant further investigation. Additionally, much of the research to date used in vitro models, and the few in vivo studies are limited by their use of immune-compromised animals. Moreover, the dual role of ASK1 in apoptosis and tumor-promoting inflammation may complicate its utility as a universal target. The net effect of ASK1 activation is strongly context-dependent. Although ASK1 activation drives apoptosis in the cancer models reviewed here, ASK1 can be pro-tumorigenic in other settings [54,55,56]. This divergence likely reflects differences in cell type, the strength and duration of ASK1 activation—with transient or moderate activation favoring adaptive survival signalling and sustained activation driving apoptosis—and the host tissue in which ASK1 is engaged. These context dependencies, together with the inflammatory and fibrotic consequences of ASK1 activation in normal tissue, underscore that any therapeutic strategy based on ASK1 activation must achieve tumor selectivity. Future research should aim to delineate the cell-type-specific effects of ASK1, identify modulators with improved selectivity, and explore its role in combination therapies.

While this review explores the anticancer potential of ASK1 activation, it should be noted that ASK1 activation is more typically considered a driver of inflammation and fibrosis in non-cancerous tissues [82]. Selonsertib (GS-4997)—the same ASK1 inhibitor used by Han et al. [62] to implicate ASK1 activation in the anticancer effects of ecliptasaponin A—has been evaluated in clinical trials for non-alcoholic steatohepatitis, pulmonary arterial hypertension, and diabetic kidney disease [83]. The fact that the principal clinical ASK1 drug is an inhibitor highlights the context-dependent role of ASK1 and raises important questions about the safety of administering an ASK1 activator to patients, as systemic ASK1 activation could have harmful effects in non-cancerous tissues. For this reason, it is critical to fully understand the mechanisms underlying ASK1 activation. Cancer cells are particularly sensitive to redox stress [34], so a drug that acts by inducing ROS generation could activate ASK1 in cancer cells while sparing normal tissue. Studies exploring ASK1 activation in cancer should therefore test their effects in comparable non-cancerous tissues to confirm the absence of adverse effects in otherwise healthy cells. Overall, targeted activation of ASK1 presents a compelling strategy for cancer therapy. Continued research into the ASK1 signalling axis in cancer may support the design and use of ASK1 activators in cancer therapy.

## Figures and Tables

**Table 1:** ASK1 mediates anticancer effects in lung cancer and mesothelioma in vitro.

Model	Treatment	Effects	Signalling	Ref.
A549 (NSCLC)	Silver nanoparticles 1–50 µg/mL 0.5–24 h	↑ Apoptosis ↓ Cell viability ↑ ROS	↑ p-ASK1 ^†^ ↑ p-JNK ^†^ ↑ p-p38 ^†^ ↑ c-Caspase-3	[57]
H1299 (NSCLC)	Indole-3-carbinol 100–700 µM 24–48 h	↓ Proliferation ↓ Colony formation ↑ Apoptosis ↑ Nuclear fragmentation ↑ Sub-G1 cell cycle arrest ↑ ROS	↑ p-ASK1 ^†^ ↓ Trx ↓ Prdx-1 ↑ p-Jnk ^†^ ↑ p-Erk ^†^ ↑ p-p38 ^†^ ↑ c-PARP ↑ c-Caspase-3 ↑ c-Caspase-7 ↑ c-Caspase-9 ↑ γ-H2AX ↑ FOXO3 ↑ Bax ↑ Bim ↓ p-Akt ^†^ ↓ Bcl-2 ↓ Bcl-xL	[58]
A549 PC9 (NSCLC)	Berberine 20–160 µM 24–72 h	↓ Cell viability ↑ Apoptosis ↑ ROS ↓ Mitochondrial membrane potential	↑ c-Caspase-3 ↑ Bax ↓ Bcl-2 ↑ p-ASK1 (Thr^845^) ↑ p-JNK ^†^ ↑ Cytosolic Cytochrome C ↓ Mitochondrial Cytochrome C	[59]
A549 (NSCLC)	CSTMP 50–150 µM 24–96 h	↓ Cell viability ↑ Apoptosis ↑ ER stress G0/G1 cell cycle arrest	↑ c-Caspase-3 ↑ c-Caspase-8 ↑ c-Caspase-9 ↑ c-PARP ↑ Bax ↓ Bcl-2 ↑ GRP78 ↑ GRP94 ↑ CHOP ↑ c-Caspase-4 ↑ c-Caspase-12 ↑ p-ASK1 ^†^ ↑ p-JNK ^†^	[60]
A549 H1975 (NSCLC)	Daidzein (300 µM) Gefitinib (10 µM) 24–48 h	↓ Cell viability ↑ Apoptosis G0/G1 cell cycle arrest ↑ ROS	↑ c-Caspase-3 ↑ c-Caspase-8 ↑ c-Caspase-9 ↑ c-PARP ↑ Bax ↓ Bcl-2 ↓ Bcl-xL ↑ Cyto-C ↑ FasL ↑ Fas ↑ FADD ↑ p-ASK1 ↑ p-JNK ↑ p53 ↑ p21 ↑ p27 ↑ p16 ↑ PTEN ↓ ABCG2 ↓ p-STAT1 ↓ p-STAT3 ↓ p-Akt ↓ p-ERK ↓ p-PI3K ↓ PI3K ↓ p-EGFR (Y1068, T845, T1092) ↓ EGFR ↓ Cyclin D1 ↓ Cyclin E	[61]
H460 H1975 (NSCLC)	Ecliptasaponin A 10–30 µM 24–48 h	↓ Cell viability ↑ Apoptosis ↑ Nuclear condensation ↑ Autophagy	↑ p-ASK1 ^†^ ↑ c-Caspase-3 ↑ c-Caspase-8 ↑ c-Caspase-9 ↑ LC3 ↑ Beclin-1 ↑ p62 ↑ p-JNK ^†^ ↑ p-Akt (Ser^473^, Thr^308^) ↓ p-ERK ^†^	[62]
A549 (NSCLC)	Denbinobin 20 µM 10 min–24 h	↑ Apoptosis ↑ ROS	↑ ASK1 activity ↑ p-JNK (Thr^183^/Tyr^185^) ↑ AP-1 ↑ p-c-Jun ^†^ ↑ Bim mRNA	[63]
H28 (Mesothelioma)	Diarachidonoylphosphoethanolamine 1–100 µM 24–48 h	↓ Cell viability ↑ Apoptosis ↑ ROS	↑ p-ASK1 (Thr^845^) ↑ p-JNK (Thr^183^/Tyr^185^) ↑ p-p38 (Thr^180^/Tyr^182^) ↓ TrxR ↓Trx	[64]

^†^ Phosphorylated residue not specified in original article; ↑ increase; ↓ decrease.

**Table 2:** ASK1 mediates anticancer effects in breast and gynecologic cancers in vitro.

Model	Treatment	Effects	Signalling	Ref.
MCF-7 MDA-MB-468 SK-BR-3	Cambogin 10 µmol/L 24–48 h	↓ Viability ↑ Apoptosis ↑ ROS	↑ p-ASK1 ↑ p-MKK4 ↑ p-MKK7 ↑ Bax/Bcl-2 ↑ AIF nuclear translocation	[65]
MDA-MB-231 MDA-MB-468 (Human breast adenocarcinoma)	Pristimerin 0.2–0.8 µM 24–72 h	↓ Viability ↓ Colony formation ↑ G1 phase arrest ↑ Apoptosis ↑ Nuclear fragmentation ↑ Cell shrinkage ↑ Autophagy ↑ ROS	↓ Cyclin D1 ↓ CDK4 ↑ p53 ↑ p21 ↑ c-Caspase-3 ↑ c-PARP ↑ LC3-II ↑ Beclin-1 ↑ p62 ↑ p-JNK ^†^ ↑ p-p38 ^†^ ↑ p-ASK1 (Thr^845^) ↓ Trx-1 activity	[66]
BGC-823 ^‡^ (Human cervical adenocarcinoma)	Curcumin 5–40 µM 24 h	↓ Viability ↑ Apoptosis ↑ ROS ↓ GSH/GSSG ratio	↑ p-ASK1 (Thr^845^) ↑ p-MKK4 (Thr^261^) ↑ p-JNK (Thr^183^/Tyr^185^)	[67]
MCF-7 (Human breast adenocarcinoma)	CLDN6 overexpression (transfection)	↓ Viability ↑ Apoptosis	↑ RIP1 ↓ p-ASK1 (Ser^967^) ↑ p-JNK ^†^ ↑ p-p38 ^†^ ↓ Bcl-2 ↑ Bax ↑ c-Caspase-3	[68]
MDA-MB-231 MCF-7 SUM190PT (Human breast adenocarcinoma)	PDK4 siRNA 48 h	↓ Viability ↑ Autophagy ↑ Ferroptosis ↑ Lipid ROS	↑ p-ASK1 ^†^ ↑ p-JNK ^†^	[69]
HeLa (Human cervical adenocarcinoma) SiHa (Human cervical squamous cell carcinoma)	α-mangostin 10–50 µM 24–48 h	↓ Viability ↑ Apoptosis ↓ MMP	↑ p-ASK1 ^†^ ↑ c-Caspase-3 ↑ c-Caspase-9 ↑ c-PARP ↑ Bax ↓ Bcl-2 ↑ Cytochrome c ↑ p-p38 ^†^	[70]
SKOV3 OVCAR3 HO8910 ^‡^ (Human ovarian adenocarcinoma)	Metformin (2–16 mM) Low glucose (0–2.5 mM) 24–48 h	↑ Metformin sensitivity ↓ Viability ↑ Apoptosis ↓ MMP ↑ ROS ↑ ER stress	↓ Bcl-2 ↑ Bax ↑c-Caspase-3 ↑ Cytochrome c ↑ Noxa ↑ p-ASK1 ^†^ ↑ p-JNK ^†^ ↑ p-AMPK ^†^ ↑ CHOP ↑ Grp78 ↑ Ub ↑ c-Caspase-4	[71]

^†^ Phosphorylated residue not specified in original article; ^‡^ HeLa misidentified cell line; ↑ increase; ↓ decrease.

**Table 3:** ASK1 mediates anticancer effects in gastrointestinal cancers in vitro.

Model	Treatment	Effects	Signalling	Ref.
SNU-213 (Human pancreatic adenocarcinoma)	Naringenin 200–600 µM 24 h	↓ Viability ↑ Apoptosis ↑ ROS	↑ ASK1 ↑ p-JNK ^†^ ↑ p-p38 ^†^ ↑ p53 ↑ p-p53 ^†^ ↓ Prdx-1 ↓ pro-PARP ↑ c-PARP ↑ Bak ↓ Bcl-XL ↑ c-Caspase-3 ↑ c-Caspase-7 ↑ c-Caspase-9 ↑ Cytochrome c	[72]
SW1990 L3.7 (Pancreatic adenocarcinoma)	Erianin 101–473 nM 48 h	↓ Viability ↓ Colony formation ↓ Migration ↑ G2/M arrest ↑ Apoptosis ↑ ROS	↑ p-ASK1 ^†^ ↑ p-JNK ^†^ ↑ p-p38 ^†^ ↓ p-AKT ↑ E-cadherin ↓ N-cadherin ↓ Vimentin ↓ β-catenin	[73]
SW480/NC SW480/NNMT HT-29/NC HT-29/shNNMT	Vanillin 2.5–4 mM 48 h	↓ Viability ↓ Colony formation ↑ Apoptosis ↑ ROS	↑ p53 ↑ c-PARP ↑ c-Caspase-3 (Asp^175^) ↑ c-Caspase-9 ↑ p-p38 (Thr^180^/Tyr^182^) ↑ p-ASK1 (Thr^845^)	[74]
HCT116 (Human colorectal carcinoma)	Adenoviral transduction (ST13 overexpression)	↑ AP-1 transcriptional activity ↓ Viability ↑ Apoptosis	↑ p-ASK1 ^†^ ↑ p-JNK ^†^ ↑ p-c-Jun ^†^ ↑ c-Caspase-3 ↑ Cytochrome c	[77]
Caco2 (Human colorectal adenocarcinoma)	Atmospheric pressure gas plasma 5, 15, and 30 s	↓ Viability ↑ Apoptosis ↑ ROS	↑ p-ASK1 (Thr^845^) ↑ p-p38 (Thr^180^/Tyr^182^) ↑ Caspase-3/-7 activity	[78]
HT29 SW480 LoVo (Human colorectal adenocarcinoma)	Anti-miR-20a (miR-20a knockdown) Cisplatin 1–30 µM 48 h	↓ Viability ↑ Cisplatin sensitivity ↑ ROS ↑ Apoptosis ↓ MMP	↑ ASK1 ↑ p-ASK1 ^†^ ↑ p-JNK ^†^ ↑ p-Bcl-2 ^†^ ↑ Cytochrome c ↑ smac/DIABLO ↑ c-Caspase-3 ↑ c-Caspase-7 ↑ c-Caspase-9	[79]

^†^ Phosphorylated residue not specified in original article; ↑ increase; ↓ decrease.

**Table 4:** ASK1 mediates anticancer effects in vivo.

Model	Treatment	Effects	Signalling	Ref.
H460 xenografted nude mice	Ecliptasaponin A 25–50 mg/kg	↓ tumor volume	-	[62]
A549 xenografted nude mice	Berberine 500 mg/kg Every other day	-	↑ p-ASK1 (Thr^845^) ↑ p-JNK ^†^ ↑ c-Caspase-3 ↑ Bax	[59]
A549 xenografted nude mice	Gefitinib 100 mg/kg Daidzein 300 mg/kg Oral gavage Twice per week for five weeks	↓ tumor volume ↓ tumor weight ↑ survival ↑ TUNEL staining	↑ p-ASK1 ^†^ ↑ p-JNK ^†^ ↑ c-Jun ↑ p-c-Jun	[61]
HeLa xenografted nude mice	α-mangostin 20–40 mg/kg Intraperitoneal injection 3 times per week	↓ tumor volume ↓ tumor weight	↑ p-ASK1 ^†^ ↑ c-Caspase-3 ↑ c-PARP ↑p-p38 ^†^	[70]
MDA-MB-231 xenografted nude mice	Pristimerin 0.5 mg/kg Intraperitoneal injection Every other day	↓ tumor volume ↓ tumor weight	↑ c-Caspase-3 ↑ LC3-II ↑ p-JNK ^†^ ↓ Trx-1 activity	[66]
LoVo xenografted nude mice	Anti-miR-20a (miR-20a knockdown) Cisplatin 10 mg/kg Twice weekly	↓ tumor volume ↓ tumor weight	↑ ASK1 ↑ p-ASK1 ^†^ ↑ p-JNK ^†^	[79]
SW1990 xenografted nude mice	Erianin (dose and route not specified)	↓ tumor volume ↓ tumor weight ↑ TUNEL staining	↑ p-JNK ^†^ ↑ p-p38 ^†^ ↑ c-Caspase-3 ↓ ki-67 ↓ p-Akt	[73]
SKOV3 xenografted nude mice	Metformin (200–600 mg/kg/day)	↓ tumor volume ↓ tumor weight	↑ p-ASK1 ^†^ ↑ CHOP ↑ Grp78 ↑c-Caspase-3	[71]

^†^ Phosphorylated residue not specified in original article; ↑ increase; ↓ decrease.

## Data Availability

No new data were created or analyzed in this study. Data sharing is not applicable to this article.
